# Characterization of Bioactive Components in Edible Algae

**DOI:** 10.3390/md18010065

**Published:** 2020-01-19

**Authors:** Leonel Pereira

**Affiliations:** Marine and Environmental Sciences Centre (MARE), Department of Life Sciences, Faculty of Sciences and Technology, University of Coimbra, 3004-517 Coimbra, Portugal; leonel.pereira@uc.pt

## Introduction

From the origin of our planet, about 4.6 billion years ago, 1.400 million years had to pass in order for an event of maximum transcendence to develop in the evolutionary history of life on Earth: the appearance of the first photosynthetic organisms, with which the biochemical machinery that maintains ecological dynamics was launched, as we know it today, both in the aquatic environment, where it was born, as well as terrestrially [1].

Photosynthesis allowed them to synthesize organic matter, the basis of the feeding of every living being, from inorganic compounds, at the same time that it was an oxygen contribution, which at that time generated a radical change in the composition of that primitive atmosphere and conditioned the next steps of evolution. Among these pioneers are the most primitive algae, with a precarious unicellular constitution that lacks a nucleus (prokaryotic cell organization). They are known as Cyanobacteria or blue-green algae, although today there is a discrepancy between systematics when considering them as bacteria or algae [2].

The next step in the evolutionary process was the formation of a complete cellular system with a true nucleus (eukaryotic cellular organization), shared by unicellular and multicellular algae, which happened just 1.4 billion years ago, with an atmosphere that was already rich in oxygen. However, 700 million years had to pass before the great algae and the first invertebrate animals were developed, and 300 million more so that, from the green algae, the first vascular plants colonized the terrestrial environment, free of living beings until that moment. The last stage in plant evolution was reached with the appearance of flowering plants (phanerogams) only 100 million years ago, which, if we compare the age of the Earth with the average life expectancy of human beings, is equivalent to two years in the life of a person [1].

## Algae as Food

Larger seaweeds, macroalgae, are a source of food ignored in the western world, while constituting an essential part of, for example, the Japanese diet. Macroalgae are, in many respects, optimal for human nutrition since they contain a prodigality of minerals, trace elements, vitamins, proteins, iodine, and essential polyunsaturated fatty acids, as well as an abundance of both soluble and insoluble dietary fibers, with few calories [3].

In the same way, macroalgae have an enormous and unexplored potential for gastronomic innovation. They can be used practically in any dish of the daily kitchen as well as haute cuisine. Considering that there are about 10,000 species worldwide of edible algae and that the genetic differences between any of these species are as large as those between plants and animals, it is clear that this splendid abundance provided by the oceans of the planet deserves greater attention from scientists and the inhabitants of the western world [4,5].

Today, the societies of western countries, so-called developed, live immersed in an illusory abundance and diversity of food. We are driven to consumption without rules or dietary care and to fast food, high in calories and unsaturated fats. This appears to be the miraculously adequate response to the hectic pace of urban life—so much so that we have even adopted the designation of ready food, or fast food, as a style and misperception of a reality, where food is seen merely as organic fuel doses to meet our most immediate energy needs. The consequences of such a diet (antagonistic to traditional slow food, more carefully prepared), where the lack of essential nutrients is evident, are obesity (and collateral diseases) as well as other diseases related to excessive intake of sugars (diabetes) and fats (arteriosclerosis), among others [3,6].

On the other hand, this illusion does not express itself with the same impact in underdeveloped or transitional countries, as seen from an economic perspective, or on those emerging economies; although in the latter, the tendency is towards their imposed consolidation rather than towards its eradication. Countries such as Brazil, with a considerable coast, face the same dilemma and have before them the path that other countries can take: where food practices can and should be adapted to local resources. Less-developed countries with an appreciable coastline, such as Angola and Mozambique, may adopt new food strategies to overcome the strong shortages still felt [7].

The question that arises at this point of consciousness is simple—what contribution or benefits can seaweed bring to the human diet in terms of food, gastronomy, or diet? The answer seems simple in the light of current knowledge—it is the exact opposite of the concept of fast food: a natural, yet wild and abundant food (with a growth rate capable of sustaining an intensive culture), capable of providing high nutritional value but at reduced caloric value. Poor in fat, seaweed has polysaccharides that behave, for the most part, as non-calorie fibers. Therefore, algae seem to be the best way to correct not only the lack of food for ingestion but also the nutritional deficiencies of the current diet worldwide (in developed, emerging, and/or underdeveloped countries) due to their wide range of essential constituents—minerals (iron and calcium), proteins (with all essential amino acids), vitamins and fiber [3]; nutrients that are absolutely necessary for human primary metabolism. They are, therefore, a guarantee of survival, which the human being, sooner or later, will resort to, now more out of whim and curiosity (thanks to some pioneering work and investments that are starting to pay off) and later, of course, to meet the demands of an explosively-growing human population that already numbers more than 7 billion people, increasingly concentrated in Asia and Africa [8].

The marine environment provides a huge source of many healthy foods including algae (seaweed–green, red, and brown macroalgae), an example of a marine product that has been part of the diet in several countries around the world. Furthermore, marine specimens are also sources of an abundance of chemicals, many of them with biological properties and therefore called bioactive compounds. These chemicals can be extracted and incorporated in several food matrices leading to new potential functional foods [6,9].

Some of the edible species referred to in this special issue have been the subject of studies related to some of their bioactive compounds, from lipids to algal phlorotannin, various phycocolloids (alginates and carrageenans), to the sulfated polysaccharides such as Ulvan and Fucoidan.

Among the species of macroalgae studied in the articles of this special issue, we find, for example, the species harvested in Indonesia and Japan, where lipid content variations were evaluated. Susanto et al. (2019) [10] concluded that the total lipid (TL) content and fatty acid composition were strongly affected by sampling location. The TL and n-3 PUFA levels tended to be higher in temperate seaweeds compared to those in tropical seaweeds. In this study were analyzed four species of edible green algae (Chlorophyta; edible seaweeds of the world): *Caulerpa lentillifera* and *Ulva reticulata*, harvested in Indonesia; *Ulva intestinalis*, *U. australis,* and *U. reticulata*, harvested in Japan; five species of red algae (Rhodophyta): *Gracilariopsis longissima*, harvested in Indonesia, *Gloiopeltis furcata, Chondrus yendoi, Mazzaella japonica* and *Chondria crassicaulis*, harvested in Japan; and six species of brown algae (Ochrophyta, Phaeophyceae): *Sargassum aquifolium,* harvested in Indonesia, *Costaria costata, Undaria pinnatifida, Saccharina japonica, Sargassum fusiforme* and *S. horneri*, harvested in Japan [5,10].

Brown (Phaeophyceae) and red (Rhodophyta) algal sulfated polysaccharides have been widely described as anticoagulant agents. However, data on green (Chlorophyta) seaweed, especially on the *Ulva* genus, are limited. In the work conducted by Adrien et al. (2019) [11], the anticoagulant activity of the ulvan extracted from *U. rigida* is evaluated. The authors of the paper conclude that the chemically-sulfated ulvan fraction could be a very interesting alternative to heparins, with different targets and high anticoagulant activity [11].

*Ecklonia cava*, an edible brown alga growing abundantly on the shores of Japan and Korea where it is consumed as part of the daily diet, is studied by the team of Oh et al. (2019) [12], which evaluated phlorotannin, phlorofuroeckol A (PFF-A), produced by this alga, and the osteoblastogenesis enhancing effects, which can be utilized against bone-remodeling imbalances and osteoporosis-related complications [12].

Natural compounds can be effective candidates for various skin diseases, especially phlorotannins extracted from seaweeds, which have interesting properties that make them useful for cosmeceutical applications. This is the theme explored by Zhen et al. (2019) [13] in an article on the effect of Eckol, extracted from edible brown seaweed *Ecklonia cava* harvested in Jeju, Korea, on the protection of dermal cells from apoptosis by inhibiting the MAPK signaling pathway. This was further reinforced by detailed investigations using MAPK inhibitors [13].

Another edible brown seaweed, *Cystoseira barbata* [5], harvested from the Romanian Black Sea coastal zone, is used in an experimental work by Trica et al. (2019) [14] for alginate extraction and a subsequent test to determine the adsorption capacity of Cu^2+^ and Pb^2+^ heavy metals [14].

The chemical, structural, and cytotoxic characterization of the invasive brown alga *Sargassum muticum* and the red alga *Osmundea pinnatifida*, collected on the Portuguese coast (Atlantic), was determined from enzymatic extractions. The team of Rodrigues et al. (2019) [15] determined from FTIR-ATR and ^1^H-NMR spectra the presence of important polysaccharide structures in the extracts, namely, fucoidans from *S. muticum* or agarans as sulfated polysaccharides from *O. pinnatifida*. No cytotoxicity against normal mammalian cells was observed, making these seaweed extracts very interesting functional ingredients, which could be explored as a food ingredient (salt replacer, nutrient vector) or nutraceutical supplement [15].

In the experimental work of Hwang et al. (2019) [16], the immune-modulatory effects of orally administrated crude fucoidan extracted from the edible brown algae *Saccharina japonica* (formerly *Laminaria japonica*) [5], collected in Taiwan, on the innate immune response, adaptive immune response, and MP antigen-stimulated immune response, was investigated. The hope of the researchers is that fucoidan, a natural food supplement, can enhance the immune responses needed for immunopotentiation and attenuate the *Mycoplasma pneumoniae* (MP) infectious disease [16].

In the work of Hofer et al. [17], seven bromophenols are isolated from a methanolic extract of the epiphytic red alga *Vertebrata lanosa*, an edible seaweed [5] collected in Brittany, France. Bioactivity of seven isolated bromophenols was tested in agar diffusion tests against *Staphylococcus aureus* and *Escherichia coli* bacteria. Three compounds showed a small zone of inhibition against both tested organisms [17].

As described by Valado and collaborators [18], changes in lipid profile constitute the main risk factor for cardiovascular diseases. The daily intake of a vegetable jelly (with carrageenan E-407) for 60 days showed a reduction in serum total cholesterol (TC) and low-density lipoprotein cholesterol (LDL-C) levels in women, allowing them to conclude that carrageenan has bioactive potential in reducing TC concentrations [18].

In the study by Cotas et al. (2019) [19], *Gigartina pistillata* (Rhodophyta) carrageenans, from specimens harvested from the western coast of Portugal, are evaluated against colorectal cancer stem cell (CSC)-enriched tumor-spheres. Carrageenans extracted from two *G. pistillata* life cycle phases have antitumor potential against colorectal cancer stem-like cells, especially the Lambda-family carrageenans extracted from the tetrasporophyte (T) phase [19].

Cho and Rhee’s review [20] focuses on research on the health benefits of consuming substances present in high concentrations in the laver, such as porphyran, vitamin B_12_, and taurine, with an evaluation of the expected effects of the consumption of these red algae. Mitigation of chemical and microbiological hazards and the adoption of new technologies to preserve and exploit the biochemical characteristics present in the *Porphyra/Pyropia* are reviewed as key strategies to further improve the quality of products based on these species (Laver/Nori).
